# Nursing students’ skills in surgical wound care improve after clinical simulation: a quasi-experiment

**DOI:** 10.1590/0034-7167-2024-0171

**Published:** 2025-09-08

**Authors:** Francisca Edinária de Sousa Borges, Francisco Gilberto Fernandes Pereira, Maria Isis Freire de Aguiar, Bruna Karen Cavalcante Fernandes, Ana Larissa Gomes Machado, Eveline Pinheiro Beserra, Joselany Áfio Caetano

**Affiliations:** I Universidade Federal do Ceará. Fortaleza, Ceará, Brazil; II Universidade Federal do Piauí. Teresina, Piauí, Brazil

**Keywords:** Simulation Training, Surgical Wound, Nursing Education, Perioperative Nursing, Nursing Students, Entrenamiento Simulado, Herida Quirúrgica, Educación en Enfermería, Enfermería Perioperatoria, Estudiantes de Enfermería

## Abstract

**Objectives:**

to assess nursing students’ skills in surgical wound care before and after clinical simulation.

**Methods:**

a quasi-experimental before-and-after study was conducted with 50 nursing students at a federal public university. Data collection occurred in three stages: assessment of students’skills before clinical simulation; application of the simulated scenario; assessment of skills after clinical simulation. Data were analyzed using McNemar’s test and Cohen’s D test.

**Results:**

clinical simulation had a positive effect on the development of nursing students’ skills in the eight dimensions of the simulated scenario, since most of the items assessed showed a significant increase in students’ performance values in the post-simulation moment.

**Conclusions:**

after clinical simulation, there was an improvement in the development of nursing students’ skills related to surgical wound care.

## RESUMEN

**Objetivos:**

evaluar las habilidades de estudiantes de enfermería en el cuidado de heridas quirúrgicas antes y después de la simulación clínica.

**Métodos:**

estudio cuasiexperimental antes y después, realizado con 50 estudiantes de enfermería de una universidad pública federal. La recolección de datos ocurrió en tres etapas: evaluación de las habilidades de los estudiantes antes de la simulación clínica; aplicación del escenario simulado; Evaluación de habilidades después de la simulación clínica. Los datos se analizaron mediante la prueba D de McNemar y Cohen.

**Resultados:**

la simulación clínica tuvo efecto positivo en el desarrollo de habilidades de los estudiantes de enfermería en las ocho dimensiones del escenario simulado, ya que la mayoría de los ítems evaluados mostraron un aumento significativo en los valores de desempeño de los estudiantes en el período post-simulación.

**Conclusiones:**

después de la simulación clínica, hubo una mejora en el desarrollo de habilidades de los estudiantes de enfermería relacionadas con el cuidado de la herida quirúrgica.

## INTRODUCTION

Clinical simulation is a dynamic, interactive and innovative method developed in a safe environment to expand the relationship between theory and practice, in addition to offering better opportunities for active learning, contributing to knowledge construction. It also allows the assessment of learning and decisions made, encouraging the development of critical reasoning and reflective discussions as well as the integration between multiple knowledge and affective, cognitive and psychosocial values^(1, 2)^.

Among the advantages highlighted in the literature, simulation is a tool that improves student satisfaction and self-confidence as well as promoting the expansion of knowledge and skills necessary for adequate management of care^(3, 4)^. Other advantages are expressed by the training of technical skills focused on teamwork, exercising leadership, clinical reasoning, decision-making, problem-solving, individual and group communication, and reducing feelings of fear and anxiety in the face of practical reality. The few disadvantages mentioned are related to simulation implementation, involving the need for investment in material resources and training of professionals^(5)^.

Among the simulation environments in nursing, the development of scenarios for chronic wound assessment and treatment stands out, since it requires adequate scientific and technological knowledge for therapeutic prescription, monitoring and prevention of complications, in addition to facilitating healing^(6)^.

Despite the growing number of investigations developed to measure wound knowledge indicators, most are directed towards the management of chronic injuries or associated complications, with simulated scenarios on surgical wounds (SWs) remaining incipient^(7)^.

Given this scenario, it is considered that SWs constitute a healthcare challenge, as they require the improvement of knowledge, skills, and attitudes for adequate management and prevention of complications^(8)^. In clinical practice, strategies aimed at surgical surveillance are still of low priority and poorly standardized, increasing the complexity of SWs and the possibility of complications. These events have a severe impact on quality of life and contribute to institutions’ financial burden, due to the need for prolonged hospitalizations and adjuvant therapies^(9)^.

In this regard, it is highlighted that measures to reduce morbidity and mortality and complications related to SWs are fundamental and can result from investments in training processes from undergraduate level, given the potential for developing the skills necessary for risk management and patient safety^(8)^.

In literature, a scenario on SWs was developed and validated by Rocha^(10)^. However, the assessment of its effect on the acquisition of skills by nursing students for managing SW care has not yet been carried out. Evidence shows that, in addition to physical, human, structural, material and financial resources, the development of knowledge and skills is necessary for the adequate management of SWs, in order to minimize the indicators of complications and morbidity and mortality^(9)^.

Therefore, the present study is justified by the need to seek, assess and propose valid, effective, safe and evidence-based strategies that are favorable to improving knowledge and technical skills for approaching the management, screening, monitoring and prevention of complications of SWs.

In this sense, the incorporation of active methodologies in health education, such as clinical simulation, represents a necessary, relevant action capable of modernizing education and contributing to the establishment of safe, effective and humanized care strategies as a mechanism to enhance learning for students and nursing professionals.

This research was conducted according to the following question: do nursing students’ skills regarding SW care change after participating in a simulated scenario developed by Rocha *et al*.^(10)^?

The publication of validated scenarios is considered to be of great importance, since clinical simulation has been widely used in health education, especially in nursing, and the validation process provides scientific rigor to the scenario. Studies of this nature can contribute to the applicability of an innovative methodology in nursing, in addition to helping future nurses to be qualified in the appropriate management of SWs, thus ensuring the safe care of future nursing professionals to society.

## OBJECTIVES

To assess nursing students’ skills in SW care before and after clinical simulation.

## METHODS

### Ethical aspects

The research met the requirements of Resolution 466/12 of the Brazilian National Health Council, since it was approved by the Research Ethics Committee of a federal university. All participants were aware of the risks and benefits and signed the Informed Consent Form.

### Study design, period and place

This is a quasi-experimental before-and-after study, carried out between November 2022 and February 2023. The research site was the School of Nursing of a federal university located in the Vale do Guaribas region in the state of Piauí.

### Population or sample; inclusion and exclusion criteria

At the time of the research, the bachelor’s degree in nursing was composed of 307 students with active enrollment, of which 77 were taking courses in the sixth (25 students), seventh (35 students) and eighth (17 students) periods. It is worth noting that only these three chunks were considered, because students had already taken the courses on nursing foundations and perioperative nursing, which guide learning regarding the care of skin injuries and their coverage. For sample delimitation, the finite sample technique by proportion was used, considering a sampling error of 5%, a confidence level of 95% and a prevalence of 54.5%^(11)^. Under these conditions, the sample was estimated at 64 participants, but only 50 were included, as they met the desired profile, 21 from the sixth period, 29 from the seventh, and 14 from the eighth.

Students regularly enrolled in undergraduate courses between the sixth, seventh and eighth semesters and aged 18 or over were included. Students with experience or previous training in courses or training with clinical simulation related to SW treatment were excluded.

### Study protocol

The study was developed in three stages: 1) Assessment of students’ skills in surgical wound management before clinical simulation; 2) Application of the simulated scenario; 3) Assessment of skills in surgical wound care after clinical simulation.

#### 1^st^ stage – Assessment of students’ skills in surgical wound management before clinical simulation

The course coordinator was asked for the email list of students in the sixth, seventh and eighth periods, to whom an invitation was sent to participate in the research and attend the skills laboratory, on previously scheduled dates and times, in groups of up to five people per time slot. At this stage, they completed a questionnaire on socio-educational data (age, sex, subjects taken related to the topic of interest, extracurricular in-depth study of content, opportunity to observe and provide care to injured patients during practical activities). They were then presented with a clinical case on SWs previously validated by experts with a general Content Validity Index equal to 1.00, in which they were to perform nursing care in an estimated time of 15 minutes. During care, three nurse researchers who were previously trained with the research protocol carried out non-participant observation guided by completing a checklist to identify the implementation of nursing skills in the care of patients with postoperative SW. This instrument was developed and validated for skin injury care, allowing for responses of the appropriate type (when the observed item was performed correctly) or inappropriate type (when the observed item was performed incorrectly). It is divided into six sections: initial observations; assessment of patients with injuries; wound and periwound skin care; referral and guidance to patients and families/caregivers; registration and documentation; final observations^(12)^.

#### 2^nd^ stage – Application of the simulated scenario

After a week of the initial internship, students attended the Simulation Center to carry out clinical simulation, according to guidelines contained in a previously validated scenario of nursing care for patients with SW^(10)^, containing the same clinical case as the first stage. The pre-briefing, briefing, round and debriefing stages were followed, mediated by the same researchers who applied the first stage. The scenario’s learning objectives were: to assess characteristics of SW and periwound skin; to identify the healing process at the incision site; to perform the appropriate dressing for SW; and to record procedures performed and wound assessment. The materials and equipment used in this stage were a high-fidelity adult simulator, a sterile dressing kit, sterile gloves, surgical mask, 0.9% saline solution, needles, sterile gauze, adhesive tape, 70% alcohol, cotton, a plastic bag for infectious waste, and a silicone-molded SW with a simple seven-point suture, which was adhered to the simulator’s abdomen.

#### 3^rd^ stage – Assessment of skills in surgical wound care after clinical simulation

The same protocol as the first stage was conducted, using the clinical case and time up to 15 minutes, since the objective of this article involves assessing skills after the intervention performed. It is worth noting that the time between the second and third phases was 30 days, according to guidelines established in another study^(13)^.

### Analysis of results, and statistics

The data were analyzed using descriptive statistics, with a measure of central tendency (mean), dispersion (standard deviation) and distribution of absolute and relative frequencies. In inferential statistics, the McNemar test was applied to verify differences in frequency of the learning level before and after the simulated intervention, conducted at a significance level of 5% as well as the assessment of the effect size by Cohen’s D (0.00-0.019 - very small; 0.20-0.49 - small; 0.50-0.79 - medium; ≥0.80 - large)^(14)^.

## RESULTS

Most participants were female (40; 75.5%), with a mean age of 22.52 (±1.24) years. All participants reported opportunities to observe and provide care to patients with wounds during undergraduate courses (100%) as well as in projects aimed at university extension (4; 21.9%).

Table 1 presents the results of assessment of students’ skills in the pre- and post-clinical simulation moments regarding scenario items: initial observations; patient and injury assessment; risk factor. Significant differences were obtained in all items assessed, from hand hygiene (<0.001), procedure guidance (0.016), all domains of clinical anamnesis, such as patient identification (0.016), assessment of emotional status (<0.001), nutritional assessment (<0.001), clinical, personal and family history, and medications in use (<0.001). Significant improvements were also identified in the assessment of local and systemic risk factors, in the use of drugs and in the identification of associated diseases.

**Table 1:** Comparison of performance in student skills related to initial observations, patient assessment and risk factors related to the surgical wound before and after clinical simulation, Picos, Piauí, Brazil, 2024

Item	Moments		*p* value*	Effect size‡
	Pre	Post		
Initial observations				
Introduces patients with surgical wounds	46(92.0)	49(98.0)	0.375	0.21
Carries out care in a closed space, ensuring privacy and comfort	47(94.0)	49(98.0)	0.625	0.13
Explains the procedure	42(84.0)	49(98.0)	0.016	0.49
Adequate language	48(96.0)	49(98.0)	1.000	0.12
Request permission and collaboration	41(82.0)	48(96.0)	0.065	0.24
Cleans hands before serving	20(40.0)	45(90.0)	<0.001	0.71
Patient and injury assessment				
Identification	42(84.0)	49(98.0)	0.016	0.28
Emotional status	26(52.0)	44(88.0)	<0.001	0.72
Clinical history and medical history	28(56.0)	45(90.0)	<0.001	0.53
Medications in use	22(44.0)	39(78.0)	<0.001	0.62
Personal habits	21(42.0)	40(80.0)	<0.001	0.31
Family history	27(54.0)	42(84.0)	<0.001	0.59
Personal hygiene	21(42.0)	38(76.0)	<0.001	0.77
Nutritional status	23(46.0)	39(78.0)	<0.001	0.32
Mobility	27(54.0)	39(78.0)	0.008	0.13
Urinary and bowel eliminations	22(44.0)	37(74.0)	<0.001	0.65
Complaint and duration of symptoms	30(60.0)	40(80.0)	0.060	0.18
History of current injury and treatments performed	26(52.0)	43(86.0)	<0.001	0.23
Identification of caregiver and institution	8(16.0)	22(44.0)	0.003	0.72
Use of forms that guide anamnesis	20(40.0)	37(74.0)	0.000	0.92
Risk factor				
Systemic	20(40.0)	38(76.0)	0.002	0.76
Local	30(60.0)	42(84.0)	0.017	0.81
Invasive procedures	5(10.0)	27(54.0)	<0.001	0.80
Use of drugs	12(24.0)	34(68.0)	<0.001	0.74
Associated diseases	20(40.0)	38(76.0)	0.001	0.66

*McNemar’s test at 5% level; SCohen’s D.

Table 2 shows the percentage of correct answers regarding students’ skills for performing physical examination, assessing SW and the presence of infection before and after clinical simulation. The verification of vital signs, as well as the use of scales to assess pain, periwound skin assessment and the presence of signs of infection, especially pain, heat, redness and odor, were items that stood out for presenting a significant frequency of correct answers after clinical simulation.

**Table 2:** Comparison of performance in student skills related to physical examination, assessment of surgical wound and presence of infection before and after clinical simulation, Picos, Piauí, Brazil, 2024

Item	Moments		*p* value*	Effect size‡
	Pre	Post		
Physical examination				
Identification of skin characteristics	39(78.0)	48(96.0)	0.022	0.11
Checks vital signs	16(32.0)	43(86.0)	<0.001	0.25
Assesses presence of pain	37(74.0)	45(90.0)	0.077	0.21
Collects detailed pain information	26(52.0)	37(74.0)	0.027	0.77
Uses scales to assess pain intensity	9(18.0)	37(74.0)	<0.001	0.87
Wound assessment				
Wound type	48(96.0)	50(100.0)	0.500	0.18
Wound location	46(92.0)	50(100.0)	0.125	0.13
Periwound skin	36(72.0)	49(98.0)	<0.001	0.83
Wound margins	36(72.0)	49(98.0)	<0.001	0.77
Exudate	43(86.0)	50(100.0)	0.016	0.48
Presence of infection				
Increase in pain	32(64.0)	45(90.0)	0.004	0.75
Periwound erythema	33(66.0)	50(100.0)	<0.001	0.87
Edema	27(54.0)	49(98.0)	<0.001	0.73
Heat	20(40.0)	50(100.0)	<0.001	0.10
Purulent exudate	40(80.0)	50(100.0)	0.002	0.59
Increased exudate level	34(68.0)	50(100.0)	<0.001	0.33
Odor	28(56.0)	50(100.0)	<0.001	0.89
Systemic manifestations	10(20.0)	22(44.0)	0.023	0.79

*McNemar’s test at 5% level; ⊠*Cohen’s D.*

In physical examination, significant differences were highlighted in students’ skills to assess pain using scales and measuring vital signs. Concerning wound assessment, the abilities to identify the margins of the injury, signs of infection and exudate were better in the post-simulation moment.

In Table 3, the skills assessed referred to wound and periwound skin care as well as referrals and guidance provided to patients and caregivers. After the intervention, significant frequencies of adjustments were observed, with emphasis on items related to cleaning the incision, wound margins and periwound skin in unidirectional movements. In the same perspective, adequate skills were observed for hand hygiene, ensuring patient comfort and privacy, use of personal protective equipment (PPE) and sterile material in fixing the dressing and disposal of contaminated material.

**Table 3:** Comparison of performance in student skills related to injury assessment and guidance to patients and family members before and after clinical simulation, Picos, Piauí, Brazil, 2024

Item	Moments		*p* value*	Effect size‡
	Pre	Post		
Wound and periwound skin care				
Cleans the incision	48(96.0)	50(100.0)	0.500	0.12
Cleans the margins	44(88.0)	50(100.0)	0.031	0.29
Removes excess saline solution	35(70.0)	46(92.0)	0.007	0.63
Mechanical cleaning of intact periwound skin	27(54.0)	50(100.0)	<0.001	0.87
Applies dressing using aseptic or sterile technique	37(74.0)	48(96.0)	0.007	0.55
Cleans hands before applying the dressing	28(56.0)	48(96.0)	<0.001	0.91
Gathers all the materials needed to apply the dressing	46(92.0)	50(100.0)	0.125	0.22
Places the injured person in a comfortable position, preserves their privacy and explains what will be done	46(92.0)	50(100.0)	0.125	0.19
Uses personal protective equipment	40(80.0)	50(100.0)	0.002	0.36
Opens the dressing material without contaminating it	44(88.0)	49(98.0)	0.125	0.17
Removes the dressing	49(98.0)	50(100.0)	1.000	0.07
Discards the removed dressing, along with the glove used in the plastic bag set aside for this purpose	49(98.0)	50(100.0)	1.000	0.14
Proceeds with cleaning the wound	50(100.0)	50(100.0)	0.250	0.18
Uses sterile gloves or tweezers to manipulate the wound	47(94.0)	50(100.0)	0.250	0.11
Occludes the wound with sterile material	45(90.0)	50(100.0)	0.063	0.38
Fixes the dressing	49(98.0)	50(100.0)	1.000	0.04
Discards the contaminated material in the bag set aside for this purpose	48(96.0)	50(100.0)	0.500	0.09
Cleans hands after applying the dressing	10(20.0)	47(94.0)	<0.001	0.48
Referral and guidance				
Hygiene	15(30.0)	19(38.0)	0.503	0.28
Hydration	10(20.0)	13(26.0)	0.629	0.18
Skin care	26(52.0)	33(66.0)	0.210	0.15
Risk factors for developing injuries	19(38.0)	38(76.0)	0.001	0.82
Clinical manifestations suggestive of worsening	14(28.0)	33(66.0)	<0.001	0.40
Prevention of complications	13(26.0)	27(54.0)	0.004	0.88
Medications used in treatment	9(18.0)	21(42.0)	0.017	0.40
Dressing care	34(68.0)	44(88.0)	0.031	0.79
Frequency of exchange and encouragement of self-care	30(60.0)	44(88.0)	0.004	0.65
Identifies the need and forwards it for assessment	9(18.0)	24(48.0)	0.004	0.43

*McNemar’s test at 5% level; ⊠*Cohen’s D.*

In the context of wound and periwound skin care, skills related to cleaning (p<0.001), drying (p=0.007), use of sterile technique (p=0.007), use of PPE (p=0.002) and hand hygiene (p<0.00Ί) also showed significant improvement with clinical simulation. Guidance on risk factors for the development of injuries, identification and prevention of signs of complications, encouragement of self-care, recording and documentation of both aspects of the injury and the procedures performed showed significant improvements.

Table 4 shows that, after simulation, the highest frequency of adjustments was concentrated in the recording of clinical assessment with identification and findings of physical examination, skills prevalent in all participants. Furthermore, the documentation of the injury type, location and characteristics, presence of exudate and odor were significant in all periods studied. In clinical assessment, the recording of pathological antecedents, as well as personal habits, medications in use, family history, personal hygiene indicators and nutritional status, improved significantly.

**Table 4:** Comparison of correct answers in student skills related to documentation and recording of care provided to patients with surgical wounds before and after clinical simulation, Picos, Piauí, Brazil, 2024

Item	Moments		*p* value*	Effect sizel
	Pre	Post		
Clinical assessment record				
Identification	44(88.0)	50(100.0)	0.031	0.36
Emotional status	20(40.0)	45(90.0)	<0.001	0.73
Clinical history and medical history	25(50.0)	45(90.0)	<0.001	0.28
Medications in use	20(40.0)	40(80.0)	<0.001	0.61
Personal habits	18(36.0)	40(80.0)	<0.001	0.25
Family history	23(46.0)	44(88.0)	<0.001	0.38
Personal hygiene	21(42.0)	37(74.0)	0.001	0.57
Nutritional status	20(40.0)	39(78.0)	<0.001	0.64
Mobility	26(52.0)	39(78.0)	0.007	0.57
Urinary and bowel elimination	17(34.0)	38(76.0)	<0.001	0.59
Complaint and duration of symptoms	30(60.0)	41(82.0)	0.013	0.49
History of current injury and treatments performed	29(58.0)	45(90.0)	<0.001	0.45
Identification of caregivers and healthcare institution attended	9(18.0)	24(48.0)	0.003	0.61
Risk factors identified	25(50.0)	45(90.0)	<0.001	0.38
Findings from physical examination	39(78.0)	46(92.0)	0.092	0.30
Makes nursing diagnosis	10(20.0)	35(70.0)	<0.001	0.77
Records the results of conduct and guidelines in subsequent assessments	25(50.0)	33(66.0)	0.115	0.19
Records assessment				
Type	46(92.0)	50(100.0)	0.125	0.16
Wound location	42(84.0)	50(100.0)	0.008	0.39
Wound duration	24(48.0)	49(98.0)	<0.001	0.82
Wound bed characteristics	34(68.0)	50(100.0)	<0.001	0.73
Periwound skin	36(72.0)	49(98.0)	<0.001	0.71
Wound margin	34(68.0)	49(98.0)	<0.001	0.63
Exudate	42(84.0)	49(98.0)	0.039	0.48
Exudate volume	34(68.0)	50(100.0)	<0.001	0.62
Odor	26(52.0)	50(100.0)	<0.001	0.87
Exposure of anatomical structures	4(8.0)	31(62.0)	<0.001	0.93
Complications during dressing application	2(4.0)	17(34.0)	<0.001	0.92
Guidance provided to family caregivers and patients	14(28.0)	39(78.0)	<0.001	0.76
Making nursing diagnosis	11(22.0)	37(74.0)	<0.001	0.69
Recording the results of procedures and guidelines in subsequent assessments	32(64.0)	37(74.0)	0.383	0.21
Final observations				
Discards disposable material used in contaminated waste, using bags intended for this purpose	45(90.0)	50(100.0)	0.063	0.59
Promotes the cleaning and disinfection of equipment and instruments used in the service	9(18.0)	35(70.0)	<0.001	0.83
Organizes the service location	35(70.0)	48(96.0)	0.001	0.75
Cleans hands after the service	10(20.0)	47(94.0)	<0.001	0.92

*McNemar’s test at 5% level; ⊠*Cohen’s D.*

Furthermore, higher adequacy rates were observed after simulation in the description of location, duration, bed characteristics, margin and periwound skin, in addition to the presence of pain or exposure of anatomical structures. Items related to documentation of complications, guidance to patients and caregivers, and nursing diagnosis also showed significant improvements. In the general observations dimension, improvements were observed in the following items: promotion of cleaning and disinfection of equipment and instruments used in care; organization of the care location; and hand hygiene after procedures.

## DISCUSSION

This study showed that nursing students’ skills in SW care were positively modified after simulation, especially regarding patient and injury assessment, wound and periwound skin care, and recording clinical assessment.

It is known that using clinical simulation as a teaching methodology allows nursing students to develop practical skills and competencies in a controlled environment, promoting patient safety and professional confidence. Thus, in nursing training, the incorporation of this teaching and learning strategy with simulated clinical scenarios is essential for the development and training of skills, competencies and professional practices^(15)^.

Among students’ skills assessed in the initial observations domain, significant differences were found in the item hand hygiene before care, with the exception of the medium effect size, which reinforces the relevance of the intervention, showing that the application of these data demonstrates the effectiveness of the strategy adopted to improve hand hygiene skills, a crucial component for preventing healthcare-related infections. It is known that hand hygiene is recognized as an effective measure for maintaining surgical wound cleanliness and reducing cross-infections. Despite this, it is still a permanent challenge in healthcare institutions, due to issues such as professional motivation and precarious physical structure^(16)^.

Therefore, it is important to highlight the impacts of simulation on future nurses’ clinical practice regarding the aforementioned item, as it is an intervention with a direct impact on reducing adverse events, health damage and mortality indicators^(17)^. Patient safety continues to be an important public health problem in Brazil, where the incidence of adverse events related to health care is high, with a national study^(18)^ demonstrating an incidence of 33.7% in Brazilian university hospitals, causing 701 additional days of hospitalization, with 58.3% of events being preventable.

In the injury assessment domain, it was found that the skills for assessing emotional and nutritional status were improved after simulation as well as clinical history, personal and family history. The effect sizes for these variables ranged from low to medium, thus highlighting the importance of educational and interventional strategies in improving the collection and management of clinical information essential for nursing practice in surgical injury care. In literature, care for patients with SW is still restricted to injury etiological assessment, with little investigation of the psychosocial impacts that the health condition can cause^(19)^.

The high prevalence of emotional reactions and psychopathological comorbidities in patients with SW, especially anxiety, depression and stress, stands out, which compromise quality of life, reduce self-care indicators and contribute to a greater occurrence of complications^(9)^. In this context, it is essential to address issues related to the psychosocial impacts of the phenomenon investigated in the nursing assessment, which was achieved based on the simulated scenario proposed in the research.

The results also demonstrated the effectiveness of simulation in developing students’ skills for performing physical examinations, assessing SW and the presence of infection. Statistical differences were observed before and after the simulation regarding cleaning the incision, wound margins and periwound skin in unidirectional movements. In the same perspective, adequate skills were observed for hand hygiene, ensuring patient comfort and privacy, use of PPE and sterile material in fixing the dressing and disposal of contaminated material.

The effect on skills, such as the use of scales to assess pain intensity, assessment of periwound skin, periwound erythema and presence of odor, was considered to be significant. It is therefore assumed that the simulated case tested offered conditions for participants to apply more systematic assessment methods that are essential for SW management, which may be a positive factor for greater adherence to care protocols that will directly reflect on the results of clinical practice.

Considering the importance of performing dressings with an appropriate technique to ensure correct healing of SW and prevent infections, these skills are essential for enabling and implementing care for both prevention and treatment of wounds^(20)^. Thus, it is considered relevant to highlight the positive performance of students after simulation for practice based on scientific evidence in search of quality in nursing care.

The care of a person with a skin injury includes a set of preventive, diagnostic and therapeutic measures that consider, in addition to the wound specificities, patients’ individuality and integrity. This perspective reinforces the need for systematization of care, through the use of guidelines, instruments, protocols and educational methodologies, such as simulation, that are favorable to clinical reasoning and appropriate decision-making^(21)^.

Failures in the systematization of nursing care are documented in the literature, as they imply inefficiency in the description of the care provided as well as the clinical condition presented by patients during nurses’ assessment^(22)^. Such actions limit the systematization of care, since the nursing process stages can be compromised.

In this regard, the results of the study in question show statistical differences in students’ skills regarding documentation and recording of care after clinical simulation. An observational study^(23)^ showed that documentation of wound treatment is essential to improve interdisciplinary communication and patient care, but is often performed inadequately and sporadically.

According to a recent study, the lack of detailed records can compromise patient safety and clinical assessment^(24)^. Clinical simulation, as a teaching methodology, helps in learning these skills by allowing students to practice proper recording in controlled situations, facilitating the internalization of critical processes for care^(25)^.

Given the contributions highlighted regarding the intervention used in the research, it is worth highlighting that clinical simulation is an essential tool in nursing education on SW care, as it allows students to practice skills in a controlled and safe environment, without risks to patients.

Additionally, simulation provides students with the opportunity to review and reflect on their procedures, which strengthens their ability to deliver effective, preventative care, thereby improving quality of care^(26)^.

As contributions to nursing education, the study promoted the improvement in the performance of skills for SW care, and proposed the adoption of clinical simulation as an educational strategy to provide active learning, the development of technical skills and competencies, and critical decision-making by nursing students in realistic scenarios.

### Study limitations

The limitation of this study refers to the sample composition that involved students between the sixth and eighth periods of the course. Thus, students in the last chunk did not participate in this investigation because they were involved in other curricular activities. Another limitation refers to the simulation laboratory structure, which, during the period of data collection, did not have equipment such as a sink, microphone, speaker, camera and monitor.

### Contributions to nursing

This study highlights simulation as a valid and effective strategy for improving nursing students’ skills in SW care. Furthermore, its potential to guide continuing education activities in different care settings is highlighted, provided that it is subjected to new validation for this purpose.

## CONCLUSIONS

It is concluded that clinical simulation through testing of a validated simulated scenario promoted improvement in the development of nursing students’ skills related to SW care. Therefore, this teaching methodology can be an option to be included as a resource for training in perioperative nursing.
