# Supplementary material for: Nursing students’ skills in surgical wound care improve after clinical simulation: a quasi-experiment
Source: Rev Bras Enferm. 2025 Sep 8;78(3):e20240171. doi: 10.1590/0034-7167-2024-0171 (PMC12419775; doi:10.1590/0034-7167-2024-0171)
Supplement: Translation

# Translation: Habilidades de estudantes de enfermagem sobre cuidado da ferida operatória melhoram após simulação clínica: quase-experimento

**DOI:** 10.1590/0034-7167-2024-0171pt

**Authors:** Francisca Edinária de Sousa Borges, Francisco Gilberto Fernandes Pereira, Maria Isis Freire de Aguiar, Bruna Karen Cavalcante Fernandes, Ana Larissa Gomes Machado, Eveline Pinheiro Beserra, Joselany Áfio Caetano

**Affiliations:** I Universidade Federal do Ceará. Fortaleza, Ceará, Brasil; II Universidade Federal do Piauí. Teresina, Piauí, Brasil

**Keywords:** Treinamento por Simulação, Ferida Operatória, Ensino em Enfermagem, Enfermagem Perioperatória, Estudantes de Enfermagem

## Abstract

**Objetivos:**

avaliar as habilidades de estudantes de enfermagem sobre cuidado da ferida operatória antes e após simulação clínica.

**Métodos:**

estudo quase-experimental do tipo antes e depois, realizado com 50 estudantes de enfermagem em uma universidade pública federal. A coleta de dados ocorreu em três etapas: avaliação das habilidades dos estudantes antes da simulação clínica; aplicação do cenário simulado; avaliação das habilidades após simulação clínica. Os dados foram analisados por meio do teste de McNemar e D de Cohen.

**Resultados:**

a simulação clínica teve efeito positivo no desenvolvimento das habilidades dos estudantes de enfermagem nas oito dimensões do cenário simulado, visto que a maioria dos itens avaliados apresentou aumento significativo nos valores de desempenho dos estudantes no momento pós-simulação.

**Conclusões:**

após simulação clínica, houve melhora no desenvolvimento de habilidade dos estudantes de enfermagem relacionada ao cuidado com a ferida operatória.

## INTRODUÇÃO

A simulação clínica constitui método dinâmico, interativo e inovador, desenvolvido em ambiente seguro para ampliar as relações entre teoria e prática, além de oferecer melhores oportunidades de aprendizagem ativa, contribuindo para construção do conhecimento. Permite ainda a avaliação da aprendizagem e das decisões tomadas, estimulando o desenvolvimento do raciocínio crítico e discussões reflexivas, assim como a integração entre os saberes múltiplos e valores afetivos, cognitivos e psicossociais^(1, 2)^.

Entre as vantagens apontadas na literatura, a simulação consta como ferramenta que melhora a satisfação e autoconfiança do discente, bem como promove a ampliação dos conhecimentos e das competências necessárias para manejo adequado da assistência^(3, 4)^. Outras vantagens são expressas pelo treino de habilidades técnicas voltadas para o trabalho em equipe, exercício da liderança, raciocínio clínico, tomada de decisão, resolução de problemas, comunicação individual e em grupo, e redução dos sentimentos de medo e ansiedade diante da realidade prática. As poucas desvantagens citadas são mencionadas em relação à implementação da simulação, envolvendo a necessidade de investimento em recursos materiais e capacitação de profissionais^(5)^.

Entre os ambientes de simulação na enfermagem, destaca-se a elaboração de cenários para avaliação e tratamento de feridas crônicas, uma vez que requer conhecimento científico e tecnológico adequado para prescrição terapêutica, monitoramento e prevenção de complicações, além de facilitar a cicatrização^(6)^.

Apesar do número crescente de investigações desenvolvidas para mensurar os indicadores de conhecimento sobre feridas, a maioria está direcionada para o gerenciamento de lesões crônicas ou complicações associadas, permanecendo como incipientes os cenários simulados sobre feridas operatórias (FOs)^(7)^.

Diante deste cenário, considera-se que as FOs constituem desafio assistencial, por exigir o aprimoramento de conhecimentos, habilidades, e atitudes para manejo adequado e prevenção de complicações^(8)^. Na prática clínica, as estratégias voltadas para vigilância cirúrgica ainda são de baixa prioridade e mal padronizadas, aumentando a complexidade das FOs e as possibilidades de complicações. Esses eventos repercutem de forma severa na qualidade de vida e contribuem para sobrecarga financeira das instituições, em virtude da necessidade de internações prolongadas e terapias adjuvantes^(9)^.

Nesse sentido, destaca-se que medidas para redução da morbimortalidade e das complicações relacionadas às FOs são fundamentais e podem decorrer de investimentos nos processos de formação desde a graduação, tendo em vista as potencialidades para o desenvolvimento das habilidades necessárias ao gerenciamento de riscos e à segurança do paciente^(8)^.

Na literatura, um cenário sobre FOs foi desenvolvido e validado por Rocha^(10)^. Entretanto, a avaliação do seu efeito na aquisição de habilidades dos discentes de enfermagem para gerenciamento do cuidado de FOs ainda não foi realizada. Evidências demonstram que, além dos recursos físicos, humanos, estruturais, materiais e financeiros, o desenvolvimento de conhecimentos e habilidades é necessário para o manejo adequado de FOs, de modo a minimizar, assim, os indicadores de complicações e morbimortalidade^(9)^.

Diante disso, justifica-se a realização do presente estudo pela necessidade de buscar, avaliar e propor estratégias válidas, eficazes, seguras e baseadas em evidências favoráveis à melhoria do conhecimento e das habilidades técnicas para abordagem no manejo, rastreio, monitorização e prevenção de complicações das FOs.

Nesse sentido, a incorporação de metodologias ativas no ensino em saúde, como a simulação clínica, representa uma ação necessária, relevante e capaz de modernizar o ensino e contribuir para o estabelecimento de estratégias assistenciais seguras, eficazes e humanizadas como mecanismo potencializador de aprendizagem de estudantes e de profissionais de enfermagem.

Esta pesquisa foi conduzida de acordo com a seguinte questão: as habilidades de estudantes de enfermagem sobre o cuidado com a FO sofrem alteração após a participação em um cenário simulado desenvolvido por Rocha *et al.* ^(10)^?

Considera-se a publicação de cenários validados de grande importância, visto que a simulação clínica vem sendo amplamente utilizada no ensino em saúde, especialmente na enfermagem, e que o processo de validação confere rigor científico ao cenário. Estudos dessa natureza podem contribuir para a aplicabilidade de uma metodologia inovadora na enfermagem, além de contribuir para que os futuros enfermeiros sejam qualificados no manejo adequado das FOs, garantindo, dessa forma, a atuação de uma assistência segura dos futuros profissionais de enfermagem à sociedade.

## OBJETIVOS

Avaliar as habilidades de estudantes de enfermagem sobre cuidado da FO antes e após simulação clínica.

## MÉTODOS

### Aspectos éticos

A pesquisa atendeu aos pressupostos da Resolução n^o^ 466/12 do Conselho Nacional de Saúde, visto que foi aprovada pelo Comitê de Ética em Pesquisa de uma universidade federal. Todos os participantes estiverem cientes dos riscos e benefícios, e assinaram o Termo de Consentimento Livre e Esclarecido.

### Desenho, período e local do estudo

Trata-se de quase-experimental do tipo antes e depois, realizado entre novembro de 2022 e fevereiro de 2023. O local da pesquisa foi a Escola de Enfermagem de uma universidade federal localizada na região do Vale do Guaribas no estado do Piauí.

### População ou amostra; critérios de inclusão e exclusão

O curso de bacharelado em enfermagem à época da pesquisa era composto por 307 discentes com matrícula ativa, dos quais 77 estavam cursando disciplinas do sexto (25 alunos), sétimo (35 alunos) e oitavo (17 alunos) períodos. Ressalta-se que apenas esses três blocos foram considerados, devido aos discentes já terem cursado as disciplinas de fundamentação de enfermagem e enfermagem perioperatória, as quais balizam a aprendizagem no tocante ao cuidado com lesões cutâneas e suas coberturas. Para delimitação amostral, utilizou-se a técnica de amostras finitas por proporção, considerando erro amostral de 5%, nível de confiança de 95% e prevalência de 54,5%^(11)^. Nessas condições, a amostra foi estimada em 64 participantes, porém apenas 50 foram incluídos, pois perfaziam o perfil desejado, sendo 21 do sexto período, 29, do sétimo, e 14, do oitavo.

Foram incluídos os discentes regularmente matriculados na graduação entre o sexto, sétimo e oitavo semestres com idade igual ou superior a 18 anos. Foram excluídos os estudantes com experiência ou capacitação prévia em cursos ou treinamentos com simulação clínica relacionados ao tratamento de FO.

### Protocolo do estudo

O estudo foi desenvolvido em três etapas: 1) Avaliação das habilidades dos estudantes no manejo da ferida operatória antes da simulação clínica; 2) Aplicação do cenário simulado; 3) Avaliação das habilidades no cuidado com a ferida operatória após simulação clínica.

#### 1ª etapa – Avaliação das habilidades dos estudantes no manejo da ferida operatória antes da simulação clínica

Solicitou-se à coordenação de curso a lista de e-mails dos estudantes do sexto, sétimo e oitavo períodos, para os quais foi enviado um convite para participação da pesquisa e comparecimento ao laboratório de habilidades, em datas e horários previamente marcados, em grupos de até cinco pessoas por horário. Nessa etapa, preencheram um questionário de dados socioeducacionais (idade, sexo, disciplinas cursadas voltadas para a temática de interesse, aprofundamento extracurricular sobre o conteúdo, oportunidade de observar e prestar cuidados ao paciente com lesão durante as atividades práticas). Em seguida, foram apresentados a um caso clínico sobre FOs previamente validado por especialistas com Índice de Validade de Conteúdo geral igual a 1,00, no qual deveriam realizar a assistência de enfermagem em um tempo estimado de 15 minutos. Durante a realização da assistência, três pesquisadores enfermeiros que foram previamente treinados com o protocolo de pesquisa realizaram observação não participante guiada pelo preenchimento de *checklist* para identificar a realização das habilidades de enfermagem no cuidado ao paciente com FO pós-operatório. Esse instrumento foi construído e validado para o cuidado com lesões de pele, permitindo respostas do tipo adequado (quando o item observado foi realizado correto) ou inadequado (quando o item observado foi incorretamente realizado). É dividido em seis sessões: observações iniciais; avaliação do paciente com lesão; cuidado com a ferida e pele perilesional; encaminhamento e orientações ao paciente e familiar/cuidador; registro e documentação; observações finais^(12)^.

#### 2ª etapa – Aplicação do cenário simulado

Após uma semana da etapa inicial, os discentes compareceram ao Centro de Simulação para realizar a simulação clínica, conforme orientações contidas em um cenário previamente validado de assistência de enfermagem a pacientes com FO^(10)^, contendo o mesmo caso clínico da primeira etapa. Foram seguidas as etapas de pré-briefing, *briefing, round* e *debriefing,* mediadas pelos mesmos pesquisadores que aplicaram a primeira etapa. Os objetivos de aprendizagem do cenário foram: avaliar as características da FO e da pele perilesional; identificar o processo de cicatrização no local da incisão; realizar o curativo adequado para a FO; e registrar procedimentos realizados e avaliação da ferida. Os materiais e equipamentos utilizados nesta etapa foram simulador adulto de alta fidelidade, *kit* para material de curativo estéril, luva estéril, máscara cirúrgica, solução fisiológica 0,9%, agulhas, gazes estéreis, esparadrapo, álcool 70%, algodão, saco plástico para resíduos infectantes, e FO moldada em silicone com sutura simples de sete pontos, a qual foi aderida ao abdômen do simulador.

#### 3ª etapa – Avaliação das habilidades no cuidado com a ferida operatória após simulação clínica

Foi conduzido o mesmo protocolo da primeira etapa, com o uso do caso clínico e tempo até 15 minutos, visto que o objetivo deste artigo envolve avaliar as habilidades após a intervenção realizada. Ressalta-se que o tempo entre a segunda e terceira fase foi de 30 dias, conforme orientações estabelecidas em outra pesquisa^(13)^.

### Análise dos resultados e estatística

Os dados foram analisados utilizando-se a estatística descritiva, com medida de tendência central (média), dispersão (desvio padrão) e distribuição de frequências absolutas e relativas. Já na estatística inferencial, foi aplicado o teste de McNemar para verificação de diferenças de frequência do nível de aprendizagem antes e após a intervenção simulada, conduzidas ao nível de significância de 5%, bem como a avaliação do tamanho de efeito pelo D de Cohen (0,00-0,019 - muito pequeno; 0,20-0,49 - pequeno; 0,50-0,79 - médio; ≥0,80 - grande)^(14)^.

## RESULTADOS

A maior parte dos participantes foi do sexo feminino (40; 75,5%), com média de idade de 22,52 (±1,24) anos. Os participantes, em sua totalidade, referiram oportunidades para observar e prestar assistência ao paciente com feridas durante as disciplinas de graduação (100%), bem como em projetos voltados para extensão universitária (4; 21,9%).

A Tabela 1 apresenta os resultados acerca da avaliação das habilidades dos estudantes nos momentos pré e pós-simulação clínica quanto aos itens do cenário: observações iniciais; avaliação do paciente e da lesão; fator de risco. Obtiveram-se diferenças significativas em todos os itens avaliados, desde a higienização das mãos (<0,001), orientação do procedimento (0,016), todos os domínios da anamnese clínica, como identificação do paciente (0,016), avaliação do estado emocional (<0,001), avaliação nutricional (<0,001), história clínica, pessoal e familiar, e medicações em uso (<0,001). Identificaram-se também melhorias significativas na avaliação dos fatores de risco locais e sistêmicos, no uso de fármacos e na identificação das doenças associadas.

**Tabela 1:** Comparação de desempenho nas habilidades discentes relacionadas às observações iniciais, avaliação do paciente e fatores de risco relacionados à ferida operatória antes e após a simulação clinica, Picos, Piauí, Brasil, 2024

Item	Momentos		Valor de p*	Tamanho de efeito‡
	Pré	Pós		
Observações iniciais				
Apresenta-se ao paciente com ferida operatória	46(92,0)	49(98,0)	0,375	0,21
Realiza atendimento em local fechado, assegura a privacidade e o conforto	47(94,0)	49(98,0)	0,625	0,13
Explica o procedimento	42(84,0)	49(98,0)	0,016	0,49
Linguagem adequada	48(96,0)	49(98,0)	1,000	0,12
Solicita a permissão e colaboração	41(82,0)	48(96,0)	0,065	0,24
Higieniza as mãos antes do atendimento	20(40,0)	45(90,0)	<0,001	0,71
Avaliação do paciente e da lesão				
Identificação	42(84,0)	49(98,0)	0,016	0,28
Estado emocional	26(52,0)	44(88,0)	<0,001	0,72
História clínica e antecedentes patológicos	28(56,0)	45(90,0)	<0,001	0,53
Medicamentos em uso	22(44,0)	39(78,0)	<0,001	0,62
Hábitos pessoais	21(42,0)	40(80,0)	<0,001	0,31
História familiar	27(54,0)	42(84,0)	<0,001	0,59
Higiene pessoal	21(42,0)	38(76,0)	<0,001	0,77
Estado nutricional	23(46,0)	39(78,0)	<0,001	0,32
Mobilidade	27(54,0)	39(78,0)	0,008	0,13
Eliminações urinária e intestinal	22(44,0)	37(74,0)	<0,001	0,65
Queixa e duração dos sintomas	30(60,0)	40(80,0)	0,060	0,18
História da lesão atual e tratamentos realizados	26(52,0)	43(86,0)	<0,001	0,23
Identificação do cuidador e instituição	8(16,0)	22(44,0)	0,003	0,72
Uso de impressos que guiam a anamnese	20(40,0)	37(74,0)	0,000	0,92
Fator de risco				
Sistêmicos	20(40,0)	38(76,0)	0,002	0,76
Locais	30(60,0)	42(84,0)	0,017	0,81
Procedimentos invasivos	5(10,0)	27(54,0)	<0,001	0,80
Uso de fármacos	12(24,0)	34(68,0)	<0,001	0,74
Doenças associadas	20(40,0)	38(76,0)	0,001	0,66

*Teste de McNemar ao nível de 5%; \D de Cohen.

A Tabela 2 apresenta a porcentagem de acertos referentes às habilidades discentes para realização do exame físico, avaliação da FO e presença de infecção antes e após simulação clínica. A verificação de sinais vitais, assim como o uso de escalas para avaliação da dor, a avaliação da pele perilesional e da presença de sinais de infecção, especialmente da dor, calor, rubor e odor, constituíram os itens que se destacaram por apresentar frequência de acertos expressiva após simulação clínica.

**Tabela 2:** Comparação de desempenho nas habilidades discentes relacionadas ao exame físico, avaliação da ferida operatória e presença de infecção antes e após simulação clínica, Picos, Piauí, Brasil, 2024

Item	Momentos		Valor de p*	Tamanho de efeito‡
	Pré	Pós		
Exame físico				
Identificação das características da pele	39(78,0)	48(96,0)	0,022	0,11
Verifica sinais vitais	16(32,0)	43(86,0)	<0,001	0,25
Avalia a presença de dor	37(74,0)	45(90,0)	0,077	0,21
Coleta informações detalhadas da dor	26(52,0)	37(74,0)	0,027	0,77
Utiliza escalas para avaliar a intensidade da dor	9(18,0)	37(74,0)	<0,001	0,87
Avaliação da ferida				
Tipo de ferida	48(96,0)	50(100,0)	0,500	0,18
Localização da ferida	46(92,0)	50(100,0)	0,125	0,13
Pele perilesional	36(72,0)	49(98,0)	<0,001	0,83
Margens da ferida	36(72,0)	49(98,0)	<0,001	0,77
Exsudato	43(86,0)	50(100,0)	0,016	0,48
Presença de infecção				
Aumento da dor	32(64,0)	45(90,0)	0,004	0,75
Eritema perilesional	33(66,0)	50(100,0)	<0,001	0,87
Edema	27(54,0)	49(98,0)	<0,001	0,73
Calor	20(40,0)	50(100,0)	<0,001	0,10
Exsudato purulento	40(80,0)	50(100,0)	0,002	0,59
Nível de exsudato aumentado	34(68,0)	50(100,0)	<0,001	0,33
Odor	28(56,0)	50(100,0)	<0,001	0,89
Manifestações sistêmicas	10(20,0)	22(44,0)	0,023	0,79

*Teste de McNemar ao nível de 5%; ‡*D de Cohen.*

No exame físico, destacaram-se diferenças significativas nas habilidades dos estudantes para avaliação da dor mediante uso de escalas e mensuração de sinais vitais. Em relação à avaliação da ferida, as habilidades para identificar as margens da lesão, os sinais de infecção e o exsudato foram melhores no momento pós-simulação.

Na Tabela 3, as habilidades avaliadas se referiram ao cuidado com a ferida e pele perilesional, assim como aos encaminhamentos e orientações prestadas aos pacientes e cuidadores. Após a intervenção, verificaram-se frequências expressivas de adequações, com destaque para os itens relacionados à limpeza da incisão, margens da ferida e pele perilesional em movimentos unidirecionais. Na mesma perspectiva, verificaram-se habilidades adequadas para higienização das mãos, garantia do conforto e privacidade ao paciente, uso de equipamento de proteção individual (EPI) e do material estéril na fixação do curativo e no descarte do material contaminado.

**Tabela 3:** Comparação de desempenho nas habilidades discentes relacionadas à avaliação da lesão e orientação a pacientes e familiares antes e após simulação clínica, Picos, Piauí, Brasil, 2024

Item	Momentos		Valor de p*	Tamanho de efeito‡
	Pré	Pós		
Cuidado com a ferida e pele perilesional				
Limpeza da incisão	48(96,0)	50(100,0)	0,500	0,12
Limpeza das margens	44(88,0)	50(100,0)	0,031	0,29
Remove o excesso de solução fisiológica	35(70,0)	46(92,0)	0,007	0,63
Limpeza mecânica da pele íntegra perilesional	27(54,0)	50(100,0)	<0,001	0,87
Realiza curativo com técnica asséptica ou estéril	37(74,0)	48(96,0)	0,007	0,55
Higieniza as mãos antes do curativo	28(56,0)	48(96,0)	<0,001	0,91
Reúne todo o material necessário à realização do curativo	46(92,0)	50(100,0)	0,125	0,22
Coloca a pessoa com lesão em posição confortável, preserva a sua privacidade e explica o que será feito	46(92,0)	50(100,0)	0,125	0,19
Faz uso de equipamento de proteção individual	40(80,0)	50(100,0)	0,002	0,36
Abre o material de curativo sem contaminá-lo	44(88,0)	49(98,0)	0,125	0,17
Remoção do curativo	49(98,0)	50(100,0)	1,000	0,07
Despreza o curativo retirado, juntamente com luva utilizada no saco plástico separado para este fim	49(98,0)	50(100,0)	1,000	0,14
Procede com a limpeza da lesão	50(100,0)	50(100,0)	0,250	0,18
Utiliza luvas estéreis ou pinças para manipular a lesão	47(94,0)	50(100,0)	0,250	0,11
Oclui a lesão com material estéril	45(90,0)	50(100,0)	0,063	0,38
Fixa o curativo	49(98,0)	50(100,0)	1,000	0,04
Despreza o material contaminado no saco separado para este fim	48(96,0)	50(100,0)	0,500	0,09
Higieniza as mãos após o curativo	10(20,0)	47(94,0)	<0,001	0,48
Encaminhamento e orientações				
Higiene	15(30,0)	19(38,0)	0,503	0,28
Hidratação	10(20,0)	13(26,0)	0,629	0,18
Cuidados com a pele	26(52,0)	33(66,0)	0,210	0,15
Fatores de risco para o desenvolvimento de lesões	19(38,0)	38(76,0)	0,001	0,82
Manifestações clínicas sugestivas do agravamento	14(28,0)	33(66,0)	<0,001	0,40
Prevenção de complicações	13(26,0)	27(54,0)	0,004	0,88
Medicações usadas no tratamento	9(18,0)	21(42,0)	0,017	0,40
Cuidados com o curativo	34(68,0)	44(88,0)	0,031	0,79
Periodicidade de troca e incentivo ao autocuidado	30(60,0)	44(88,0)	0,004	0,65
Identifica a necessidade e encaminha para avaliação	9(18,0)	24(48,0)	0,004	0,43

*Teste de McNemar ao nível de 5%; ‡*D de Cohen.*

No contexto do cuidado com a ferida e pele perilesional, as habilidades relacionadas à limpeza (p<0,001), secagem (p=0,007), uso da técnica estéril (p=0,007), uso de EPI (p=0,002) e higienização das mãos (p<0,001) também apresentaram melhora significativa com a simulação clínica. Orientações quanto aos fatores de risco para o desenvolvimento de lesões, identificação e prevenção de sinais de complicação, incentivo ao autocuidado, registro e documentação tanto dos aspectos da lesão quanto dos procedimentos realizados apresentaram melhoras significativas.

Na Tabela 4, verifica-se que, após a simulação, a maior frequência de adequações se concentrou no registro da avaliação clínica com identificação e achados do exame físico, habilidades prevalentes na totalidade dos participantes. Ainda, a documentação do tipo, localização e características da lesão, presença de exsudato e odor foram expressivos em todos os períodos estudados. Na avaliação clínica, o registro dos antecedentes patológicos, assim como dos hábitos pessoais, medicações em uso, histórico familiar, indicadores de higiene pessoal e estado nutricional, melhorou significativamente.

**Tabela 4:** Comparação de acertos nas habilidades discentes relacionadas à documentação e registro da assistência prestada ao paciente com ferida operatória antes e após simulação clínica, Picos, Piauí, Brasil, 2024

Item	Momentos		Valor de p*	Tamanho de efeito‡
	Pré	Pós		
Registro da avaliação clínica				
Identificação	44(88,0)	50(100,0)	0,031	0,36
Estado emocional	20(40,0)	45(90,0)	<0,001	0,73
História clínica e antecedentes patológicos	25(50,0)	45(90,0)	<0,001	0,28
Medicamentos em uso	20(40,0)	40(80,0)	<0,001	0,61
Hábitos pessoais	18(36,0)	40(80,0)	<0,001	0,25
História familiar	23(46,0)	44(88,0)	<0,001	0,38
Higiene pessoal	21(42,0)	37(74,0)	0,001	0,57
Estado nutricional	20(40,0)	39(78,0)	<0,001	0,64
Mobilidade	26(52,0)	39(78,0)	0,007	0,57
Eliminações urinária e intestinal	17(34,0)	38(76,0)	<0,001	0,59
Queixa e duração dos sintomas	30(60,0)	41(82,0)	0,013	0,49
História da lesão atual e tratamentos realizados	29(58,0)	45(90,0)	<0,001	0,45
Identificação do cuidador e instituição de saúde que frequenta	9(18,0)	24(48,0)	0,003	0,61
Fatores de risco identificados	25(50,0)	45(90,0)	<0,001	0,38
Achados do exame físico	39(78,0)	46(92,0)	0,092	0,30
Faz o diagnóstico de enfermagem	10(20,0)	35(70,0)	<0,001	0,77
Registra o resultado das condutas e orientações nas avaliações subsequentes	25(50,0)	33(66,0)	0,115	0,19
Registra a avaliação				
Tipo	46(92,0)	50(100,0)	0,125	0,16
Localização da ferida	42(84,0)	50(100,0)	0,008	0,39
Duração da ferida	24(48,0)	49(98,0)	<0,001	0,82
Características do leito da ferida	34(68,0)	50(100,0)	<0,001	0,73
Pele perilesional	36(72,0)	49(98,0)	<0,001	0,71
Margem da ferida	34(68,0)	49(98,0)	<0,001	0,63
Exsudato	42(84,0)	49(98,0)	0,039	0,48
Volume do exsudato	34(68,0)	50(100,0)	<0,001	0,62
Odor	26(52,0)	50(100,0)	<0,001	0,87
Exposição de estruturas anatômicas	4(8,0)	31(62,0)	<0,001	0,93
Intercorrências durante a realização do curativo	2(4,0)	17(34,0)	<0,001	0,92
Orientações fornecidas ao familiar cuidador e ao paciente	14(28,0)	39(78,0)	<0,001	0,76
Faz o diagnóstico de enfermagem	11(22,0)	37(74,0)	<0,001	0,69
Registra o resultado das condutas e orientações nas avaliações subsequentes	32(64,0)	37(74,0)	0,383	0,21
Observações finais				
Despreza material descartável usado no lixo contaminado, utilizando sacos destinados para este fim	45(90,0)	50(100,0)	0,063	0,59
Promove a limpeza e desinfecção de equipamentos e instrumentais utilizados no atendimento	9(18,0)	35(70,0)	<0,001	0,83
Organiza o local do atendimento	35(70,0)	48(96,0)	0,001	0,75
Higieniza as mãos após o atendimento	10(20,0)	47(94,0)	<0,001	0,92

*Teste de McNemar ao nível de 5%; ‡*D de Cohen.*

Ainda, maiores índices de adequação foram verificados após simulação na descrição da localização, duração, características do leito, margem e pele perilesional, além da presença de dor ou da exposição de estruturas anatômicas. Os itens relacionados à documentação de intercorrências, orientações ao paciente e cuidador, e diagnóstico de enfermagem também apresentaram melhorias significativas. Na dimensão observações gerais, foram observadas melhorias nos seguintes itens: promoção da limpeza e desinfecção de equipamentos e instrumentais utilizados no atendimento; organização do local de atendimento; e higienização das mãos após procedimento.

## DISCUSSÃO

Este estudo mostrou que as habilidades dos estudantes de enfermagem sobre cuidado da FO foram modificadas positivamente após a realização da simulação, em especial no que se refere à avaliação do paciente e da lesão, ao cuidado com a ferida e a pele perilesional, e ao registro da avaliação clínica.

Sabe-se que utilizar a simulação clínica como metodologia de ensino permite que os estudantes de enfermagem desenvolvam habilidades e competências práticas em um ambiente controlado, promovendo a segurança do paciente e confiança profissional. Assim, na formação em enfermagem, a incorporação dessa estratégia de ensino e aprendizagem com cenários clínicos simulados é fundamental para desenvolvimento e treino de habilidades, competências e práticas profissionais^(15)^.

Entre as habilidades dos estudantes avaliadas no domínio observações iniciais, diferenças significativas foram verificadas no item higienização das mãos antes do atendimento, com ressalva para o tamanho de efeito considerado médio, dado que reforça a relevância da intervenção, evidenciando que a aplicação desses dados demonstra a efetividade da estratégia adotada para melhorar a habilidade de higienização das mãos, um componente crucial para a prevenção de infecções relacionadas à assistência à saúde. Sabe-se que a higienização das mãos é reconhecidamente uma medida eficaz para manutenção da limpeza da ferida cirúrgica e redução de infecções cruzadas. Apesar disso, ainda é um desafio permanente nas instituições de saúde, devido às questões como motivação dos profissionais e estrutura física precária^(16)^.

Assim, torna-se relevante salientar os impactos da simulação na prática clínica dos futuros enfermeiros quanto ao item retrocitado, por ser uma intervenção com impacto direto na redução dos eventos adversos, danos à saúde e indicadores de mortalidade^(17)^. A segurança do paciente continua sendo um importante problema de saúde pública no Brasil, cuja incidência de eventos adversos relacionados à assistência à saúde é alta, sendo demonstrada em estudo nacional^(18)^ incidência de 33,7% em hospitais universitários brasileiros, causando 701 dias adicionais de internação, sendo 58,3% dos eventos evitáveis.

No domínio avaliação da lesão, verificou-se que as habilidades para avaliação do estado emocional e nutricional foram melhoradas após a simulação, bem como da história clínica, antecedentes pessoais e familiares. Os tamanhos de efeito nessas variáveis oscilaram entre baixo e médio, destacando, assim, a importância de estratégias educativas e interventivas em melhorar a coleta e o manejo de informações clínicas essenciais para a prática de enfermagem no cuidado às lesões operatórias. Na literatura, a assistência ao paciente com FO ainda é restrita à avaliação etiológica da lesão, sendo pouco investigados os impactos psicossociais que a condição de saúde pode acarretar^(19)^.

Destaca-se, assim, a alta prevalência de reações emocionais e comorbidades psicopatológicas em pacientes com FO, sobretudo de ansiedade, depressão e estresse, que comprometem a qualidade de vida, reduz os indicadores de autocuidado e contribui para maior ocorrência de complicações^(9)^. Nesse contexto, torna-se imprescindível abordar questões relacionadas aos impactos psicossociais do fenômeno investigado na avaliação de enfermagem, o que foi alcançado a partir do cenário simulado proposto na pesquisa.

Os resultados demonstraram ainda a efetividade da simulação no desenvolvimento de habilidades dos estudantes para realização do exame físico, da avaliação da FO e da presença de infecção. Diferenças estatísticas foram observadas antes e depois da simulação quanto à limpeza da incisão, margens da ferida e pele perilesional em movimentos unidirecionais. Na mesma perspectiva, verificaram-se habilidades adequadas para higienização das mãos, garantia do conforto e privacidade ao paciente, uso de EPI e do material estéril na fixação do curativo e no descarte do material contaminado.

Foi considerado grande o efeito nas habilidades, como uso de escalas para avaliar a intensidade da dor, avaliação da pele perilesional, eritema perilesional e presença de odor. Presume-se, portanto, que o caso simulado testado ofereceu condições para os participantes aplicarem métodos de avaliação mais sistemáticas que são indispensáveis ao manejo da FO, o que pode ser um fator positivo para maior adesão a protocolos de cuidados que refletirão diretamente nos resultados da prática clínica.

Considerando a importância de realizar curativo com técnica adequada para garantir a cicatrização correta da FO e prevenir infecções, essas habilidades são primordiais para viabilização e implementação de cuidados tanto para a prevenção quanto para tratamento de feridas^(20)^. Assim, considera-se relevante destacar o desempenho positivo dos estudantes após a simulação para a prática baseada em evidências científicas em busca da qualidade da assistência de enfermagem.

O cuidado da pessoa com lesão cutânea abrange um conjunto de medidas preventivas, diagnósticas e terapêuticas que considerem, além das especificidades da ferida, a individualidade e integralidade do paciente. Essa perspectiva reforça a necessidade da sistematização do cuidado, mediante a utilização de diretrizes, instrumentos, protocolos e metodologias educacionais, como a simulação, que sejam favoráveis ao raciocínio clínico e à tomada adequada de decisão^(21)^.

Falhas na sistematização dos cuidados de enfermagem são documentadas na literatura, pois implicam ineficiência na descrição do cuidado prestado, assim como da condição clínica apresentada pelo paciente durante a avaliação do enfermeiro^(22)^. Tais ações são limitantes da sistematização do cuidado, uma vez que as etapas do processo de enfermagem podem ser comprometidas.

Nesse aspecto, os resultados do estudo em tela mostram diferenças estatísticas nas habilidades dos estudantes acerca da documentação e registro da assistência após simulação clínica. Estudo observacional^(23)^ mostrou que a documentação do tratamento de feridas é essencial para melhorar a comunicação interdisciplinar e o atendimento ao paciente, mas muitas vezes é realizada de forma inadequada e esporádica.

Segundo estudo recente, a ausência de registros detalhados pode comprometer a segurança do paciente e avaliação clínica^(24)^. A simulação clínica, como metodologia de ensino, auxilia na aprendizagem dessas habilidades ao permitir que estudantes pratiquem o registro adequado em situações controladas, facilitando a internalização de processos críticos para o cuidado^(25)^.

Diante das contribuições destacadas quanto à intervenção utilizada na pesquisa, salienta-se que a simulação clínica é uma ferramenta essencial no ensino de enfermagem sobre o cuidado de FO, pois permite que os estudantes pratiquem habilidades em ambiente controlado e seguro, sem riscos para o paciente.

Além disso, a simulação proporciona aos alunos a oportunidade de revisar e refletir sobre seus procedimentos, o que fortalece sua capacidade de realizar cuidados eficazes e preventivos, melhorando, assim, a qualidade do atendimento^(26)^.

Como contribuições para o ensino de enfermagem, o estudo promoveu a melhora no desempenho de habilidades para o cuidado de FO, e propôs a adoção da simulação clínica como estratégia educacional para proporcionar a aprendizagem ativa, o desenvolvimento de habilidades e competências técnicas, e a tomada de decisões críticas dos estudantes de enfermagem em cenários realistas.

### Limitações do estudo

A limitação do estudo refere-se à composição amostral que envolveu discentes entre o sexto e oitavo período de curso. Assim, estudantes do último bloco não participaram desta investigação por estarem envolvidos em outras atividades curriculares. Outra limitação refere-se à estrutura do laboratório de simulação, que, no período da coleta de dados, não possuía equipamentos como pia, microfone, caixa de som, câmera e monitor.

### Contribuições para a área da enfermagem

Este estudo evidencia a simulação como estratégia válida e efetiva para melhoria das habilidades de estudantes de enfermagem acerca dos cuidados com a FO. Ainda, destaca-se seu potencial para nortear atividades de educação permanente nos diferentes cenários assistenciais, desde que seja submetido a uma nova validação para essa finalidade.

## CONCLUSÕES

Conclui-se que a simulação clínica por meio da testagem de um cenário simulado validado promoveu melhora no desenvolvimento de habilidade dos estudantes de enfermagem relacionada ao cuidado com a FO. Portanto, essa metodologia de ensino pode ser uma opção a ser incluída como recurso para formação na área da enfermagem perioperatória.
